# Supplementary material for: A structural and functional subdivision in central orbitofrontal cortex
Source: Nat Commun. 2022 Jun 24;13:3623. doi: 10.1038/s41467-022-31273-9 (PMC9232485; doi:10.1038/s41467-022-31273-9)
Supplement: Supplementary file 1 — Supplementary Information [file 41467_2022_31273_MOESM1_ESM.pdf]

Supplementary material

Neuroanatomy

A second injection, M6FR, targeted the PCC sulcus. There was anatomical connectivity with cOFC*m*, although it was less specific than observed in M1FR (**Supplementary Figure 1**).

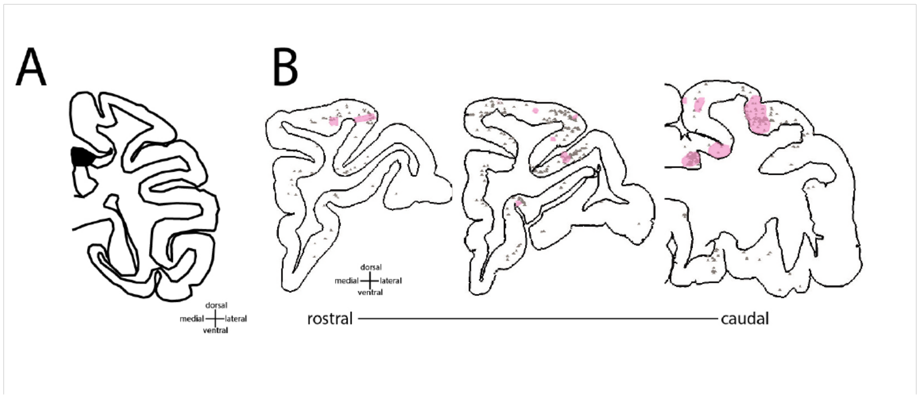

Supplementary Figure 1: Anatomical connectivity in prefrontal cortex following an injection in the PCC sulcus. A. Injection site in PCC **B.** Terminal field labeling shown in pink; retrograde labeling shown as gray dots.

Behavior in the gambling task

Two subjects combined. We examined the behavior of two male macaque subjects (*Macaca mulatta*, subjects P and S) performing a well-studied two-option risky choice task (Strait et al., 2014). The data and results we present here have not been published before, but qualitatively replicate our past findings. Specifically, behavioral data indicate that subjects understood the key elements of the task. They preferred offers with the larger expected value on 73.10% of the trials (for individual subjects, see below). This proportion is significantly higher than expected by chance (p<0.001, binomial test). It is also quantitatively similar to numbers we have found using the same task in other subjects (Strait et al., 2014; Strait et al., 2015). Subjects' willingness to choose an offer varied as a function of the difference in values between the two offers (**Supplementary Figure 2A**). Both subjects slightly preferred offer 2, although the size of the effect was small; choosing offer 1 46.90% of the time). Note that these behavioral results are restricted to trials in which our physiological recordings met criteria for analysis. Data collected in other sessions were not noticeably different (data not shown).

Each individual subject. Behaviors of each individual subject closely resembled those of two subjects combined as reported in the main text. Subject P preferred offers with the larger expected value on 73.35% of the trials (**Supplementary Figure 2B**). This proportion is significantly higher than expected by chance (479 out of 653, p<0.001, binomial test). P shifted choices from offer 1 to offer 2 as

the expected value difference of offer 1 minus offer 2 decreased, even with a slight bias against offer 1 (psychometrics function slightly shifted towards right, choosing offer 1 43.64% of the time). Subject S preferred offers with the larger expected value on 72.39% of the trials (**Supplementary Figure 2B**). This proportion is significantly higher than expected by chance (367 out of 507, $p<0.001$, binomial test). S shifted their choice from offer 1 to offer 2 as the expected value difference of offer 1 minus offer 2 decreased, even with a slight bias against offer 1 (psychometrics function slightly shifted towards right, choosing offer 1 45.18% of the time).

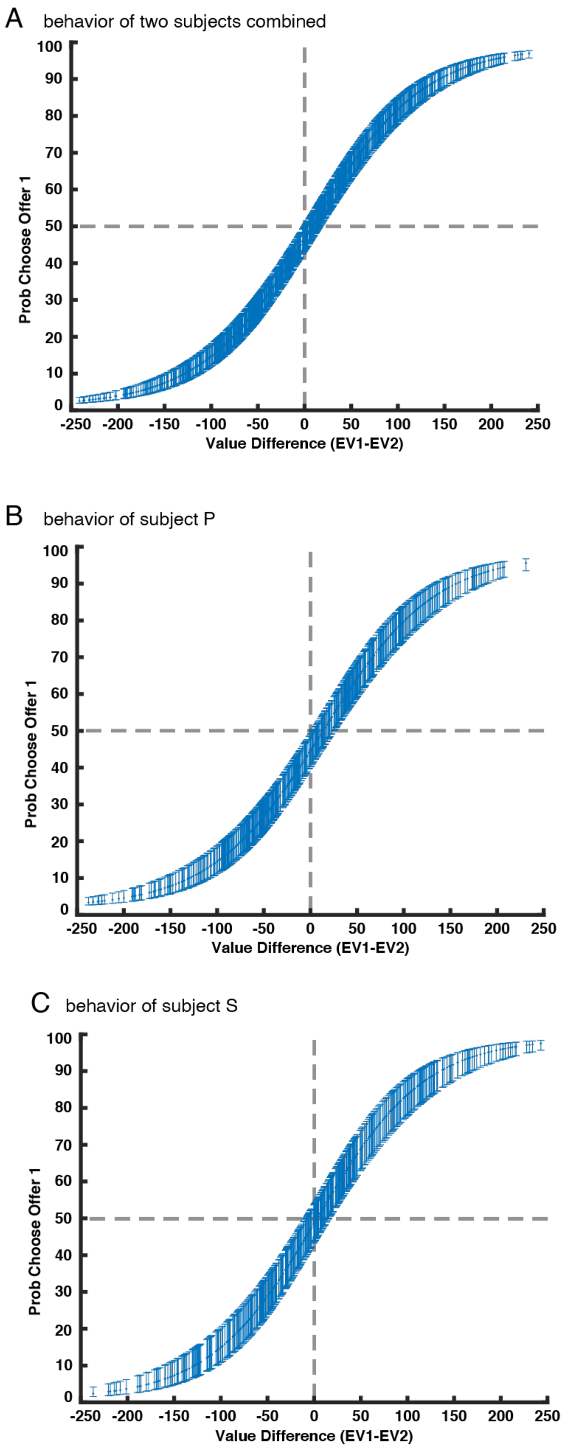

Supplementary Figure 2: Behavior in the gambling task. A. Choices of two subjects combined. **B.** Choices of subject P. **C.** Choices of subject S. EV, expected value (see **Methods**). Gray dotted lines represent visual reference for value 0 on X axis and value 50 on Y axis. Error bars on the fitted sigmoidal function represents 95% confidence interval from the

model estimation. **A-C.** Data are presented as logistic regression model estimation of the mean probability of choosing offer 1 +/- 95% confidence interval.

Functional connectivity

We first characterized the local field potentials in each of the cOFC*m*, cOFC*l*, and PCC regions. With multitaper spectral analyses, we show that power peaked around 10 Hz in cOFC*m* and cOFC*l*, and around 10 and 20 Hz in PCC (**Supplementary Figure 3A-C**). It also shows that our notch filter effectively removed power around 60 Hz.

The higher coherence in the cOFCm_{spk} - PCC_{lfp} circuit than in cOFCl_{spk} - PCC_{lfp} circuit was also observed within all specific bands that we tested: the delta (0.5-5 Hz) frequency band (z=2.53, p=0.012), the theta (5-10 Hz) band (z=3.55, p<0.001), the alpha (10-15 Hz) band (z=3.83, p<0.001), the beta (15-30 Hz) band (z=4.38, p<0.001), and the gamma (30-100 Hz) band (z=5.51 p<0.001). Comparing coherence across all five frequency bands within each circuit, during offer epoch, there was no significant difference among frequency bands within either the cOFCm_{spk} - PCC_{lfp} (χ^2=3.95, p=0.413, Kruskal-Wallis test) or the cOFCl_{spk} - PCC_{lfp} (χ^2=2.28, p=0.685, Kruskal-Wallis test) circuit.

We observed similar results in the choice epoch. We also found higher coherence in cOFCm_{spk} - PCC_{lfp} than in cOFCl_{spk} - PCC_{lfp} circuit, within the theta (z=1.98, p=0.047), the alpha (z=3.14, p=0.002), and the gamma (z=3.73, p<0.001) bands, although not within the delta (z=1.10, p=0.271) or the beta (z=1.41, p=0.159) bands. Comparing the coherence across all five frequency bands within each circuit, during choice epoch, there was no significant difference among frequency bands within either cOFCm_{spk} - PCC_{lfp} (χ^2=1.81, p=0.771, Kruskal-Wallis test) or cOFCl_{spk} - PCC_{lfp} (χ^2=2.53, p=0.640, Kruskal-Wallis test).

Finally, we observed the same general pattern during the outcome and the reward epochs. We found higher coherence cOFCm_{spk} - PCC_{lfp} than in cOFCl_{spk} - PCC_{lfp} circuit, within the delta (z=3.36, p<0.001), the theta (z=2.87, p=0.004), the alpha (z=3.70, p=0.002), and the gamma (z=2.05, p=0.040) bands, although not within the beta (z=1.27, p=0.204) band. Comparing the overall coherence across all five frequency bands within each circuit, during reward epoch, there was significant difference among frequency bands within cOFCm_{spk} - PCC_{lfp} (χ^2=14.32, p=0.006, Kruskal-Wallis test with Tukey-Kramer multiple comparison) circuit. Specifically, within cOFCm_{spk} - PCC_{lfp}, the coherence in the beta band was significantly lower than that in the theta band (p=0.021) and that in the alpha band (p=0.009). There was also a significant difference among frequency bands within cOFCl_{spk} - PCC_{lfp} circuit (χ^2=17.15, p=0.002, Kruskal-Wallis test with Tukey-Kramer multiple comparison). Specifically, within cOFCl_{spk} - PCC_{lfp}, the coherence in the alpha band was significantly lower than that in the theta band (p=0.007) and that in the gamma band (p=0.029).

Together, we found greater coherence in cOFCm_{spk} - PCC_{lfp} than in cOFCl_{spk} - PCC_{lfp}, suggesting stronger functional connectivity. This pattern of enhanced coherence was not found in the reverse direction (that is, in the PCC_{spk} - cOFCm_{lfp}, PCC_{spk} - cOFCl_{lfp} circuits; **Supplementary Figure 3D-J**).

We further compared the broadband spike-field coherence in the reverse direction to that reported in the main text. We found significantly higher broadband coherence in cOFCm_{spk} - PCC_{lfp} than PCC_{spk} - cOFCm_{lfp} ($z=4.83$, $p<0.001$, Wilcoxon signed rank test, **Supplementary Figure 3D**). The broadband coherence was also higher in cOFCl_{spk} - PCC_{lfp} than in PCC_{spk} - cOFCl_{lfp} ($z=2.90$, $p=0.004$, Wilcoxon signed rank test, **Supplementary Figure 3E**). We also found significantly higher broadband coherence in PCC_{spk} - cOFCm_{lfp} than in PCC_{spk} - cOFCl_{lfp} ($z=2.76$, $p=0.006$, Wilcoxon signed rank test; **Supplementary Figure 3D-E**) but no significant differences in broadband coherence between cOFCm_{spk} - cOFCl_{lfp} and cOFCl_{spk} - cOFCm_{lfp} ($z=0.15$, $p=0.883$, Wilcoxon signed rank test; **Supplementary Figure 3F-G**).

Spike-field coherence is theorized to capture long-range input from the spiking region to the field region. Our results suggest that the enhanced synchronization for cOFC*m*-PCC could be dominated by cOFC*m*'s input to influencing PCC local neurocomputation.

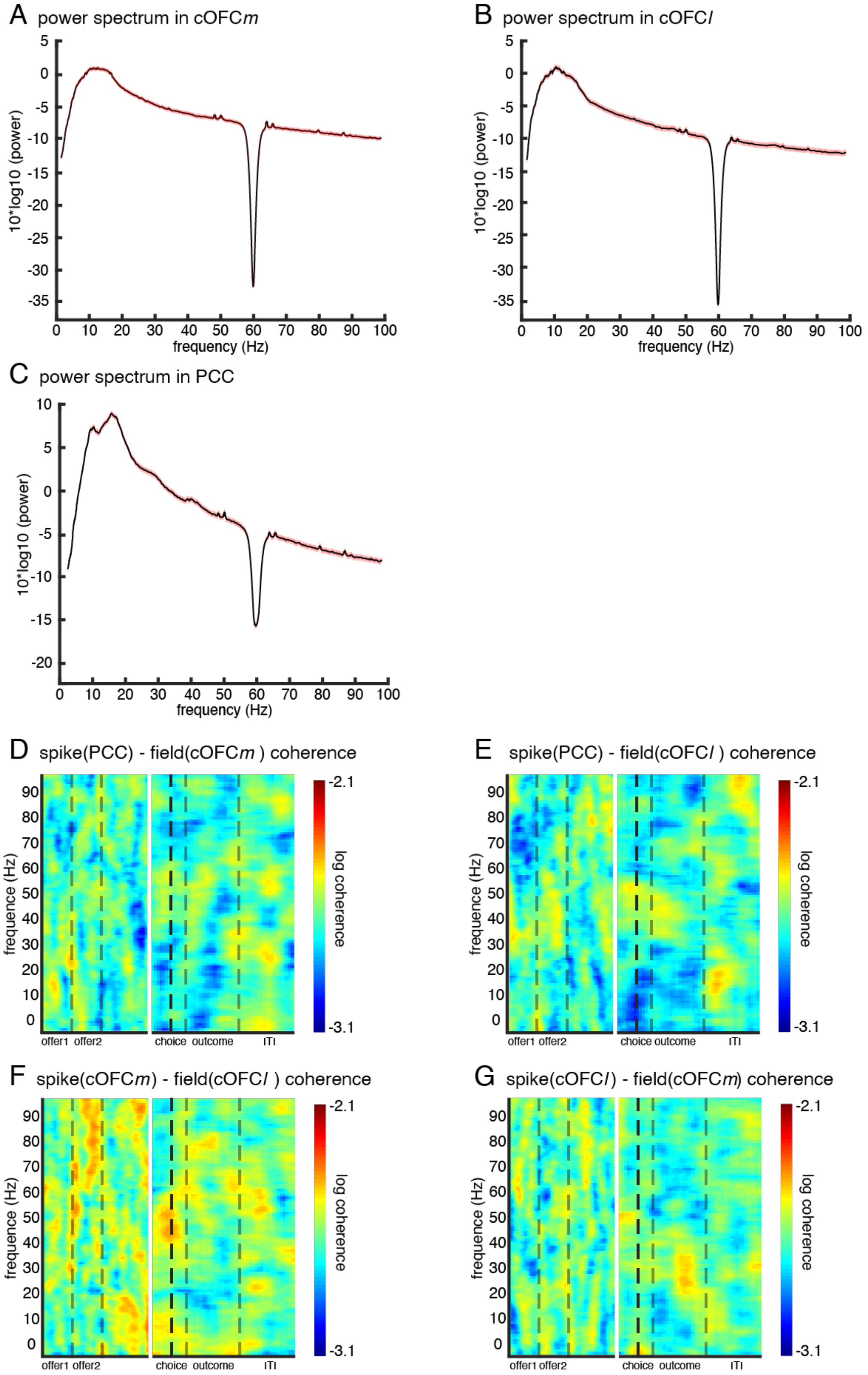
A power spectrum in cOFCm
10*log10 (power)
frequency (Hz)
B power spectrum in cOFCl
10*log10 (power)
frequency (Hz)
C power spectrum in PCC
10*log10 (power)
frequency (Hz)
D spike(PCC) - field(cOFCm) coherence
frequence (Hz)
offer1 offer2
choice outcome
ITI
log coherence
-2.1
-3.1
E spike(PCC) - field(cOFCl) coherence
frequence (Hz)
offer1 offer2
choice outcome
ITI
log coherence
-2.1
-3.1
F spike(cOFCm) - field(cOFCl) coherence
frequence (Hz)
offer1 offer2
choice outcome
ITI
log coherence
-2.1
-3.1
G spike(cOFCl) - field(cOFCm) coherence
frequence (Hz)
offer1 offer2
choice outcome
ITI
log coherence
-2.1
-3.1

Supplementary Figure 3: Supplementary Functional Connectivity. A-C. Power spectrum in cOFC***m*** (A), in cOFC***l*** (B), and in PCC (C). X axis: frequency (Hz). Y axis: power transformed with a 10 × 10 function. Black line: mean power across channel and across trials. Red shaded area: 95% confidence interval. **D-G.** Spike-field coherence. X axis: time in a trial. Y axis: frequency. Color: strength of spike-field coherence on log10 scale. The warmer the color, the higher the coherence. Data from the first half of the trial (offer period) was aligned at offer 1 onset. Data from the second half of the trial (choice period) was aligned at Choice execution. (D) PCC$_{spk}$-cOFC***m***$_{lfp}$ coherence. (E) PCC$_{spk}$- cOFC***l***$_{lfp}$ coherence. (F) cOFC***m***$_{spk}$- cOFC***l***$_{lfp}$ coherence. (G) cOFC***l***$_{spk}$cOFC***m***$_{lfp}$ coherence.

Greater mutual information between cOFC*m*-PCC and cOFC*l* -PCC circuits

We found that the cOFC*m*-PCC circuit shared higher level of mutual information than cOFC*l* -PCC (z=17.47, p<0.001, Wilcoxon signed rank test). Specifically, the cOFC*m*-PCC circuit shared 7.44×10^{-4} bits of information per channel, while the cOFC*l* -PCC circuit shared 6.72×10^{-4} bits per channel. This difference was observed during the offer 1 epoch (z=8.81, p<0.001), during the offer 2 epoch (z=8.34, p<0.001), during the choice epoch (z=9.42, p<0.001), and during the reward epoch (z=8.23, p<0.001). The difference was not observed during the inter-trial interval epoch (ITI, z=0.71, p=0.479).

Encoding of offer, choice, and outcome

We examined neural encoding of task parameters and behavior in cOFC*m*, cOFC*l*, and PCC using the proportion of neurons, the encoding strength, and the latency to peak encoding strength (**Methods**). All three regions encoded offer and outcome values with similar proportion of neurons, encoding strength, and latencies.

During the presentation of the first offer, 18.18% (n=8/44, p=0.001, binomial test) of cOFC*m* neurons, 16.67% (n=9/54, p=0.001) of cOFC*l* neurons, and 13.62% (n=29/213, p<0.001) of PCC neurons encoded the value of offer 1. These proportions were not detectably different from one another (χ^2=0.79, df=2, p=0.675, Chi-square test).

We used the t-statistics of each predictor in a multiple regression model as a measure of encoding strength (**Methods**). Encoding strength of offer 1 value at the population level was not different among cOFC*m*, cOFC*l*, and PCC (χ^2=1.67, p=0.434, Kruskal-Wallis test). We then assessed response latencies using a generalized linear model with Gamma distribution (Bishop, 2006; MacKay, 2003). For the latency analysis, we used all neurons, because many neurons in a population can show encoding of task

variables without passing statistical significance; considering all neurons improves accuracy. Among all neurons, the encoding strength of offer 1 value peaked at 290 ms in cOFC*m*, 235 ms in cOFC*l*, and 240 ms in PCC, after offer 1 onset. We then used the distributions of single-neuron latencies to assess statistical significance; by this method, these latencies were not significantly different from one another (F=1.39, p=0.251, GLM with Gamma distribution; **Methods**).

During the outcome epoch, 34.09% (n=15/44, p<0.001, binomial test) of cOFC*m* neurons, 35.19% (n=19/54, p<0.001, binomial test) of cOFC*l* neurons, and 52.58% (n=112/213, p<0.001, binomial test) of PCC neurons encoded the value of received outcome. The proportion of such neurons in PCC was significantly higher than those of cOFC*m* and cOFC*l* (χ^2=8.63, df=2, p=0.013, Chi-square test, cf. Hayden et al., 2008). The encoding strength of outcome value at the population level was significantly higher in PCC than both cOFC*m* and cOFC*l* (χ^2=9.83, p=0.007, Kruskal-Wallis test with Tukey-Kramer multiple comparison). The encoding of outcome value peaked around 275 ms in cOFC*m*, 360 ms in cOFC*l*, and 450 ms in PCC, after reward onset. These latencies were not significantly different from one another (F=1.30, p=0.275, GLM with Gamma distribution).

All three regions encoded chosen option (offer 1 vs. 2) and chosen location (left vs. right). However, cOFC*m* encoded the chosen option with shorter latency than both cOFC*l* and PCC. PCC, not cOFC*m* nor cOFC*l*, showed a higher proportion of neurons encoding chosen location than chosen option. PCC and cOFC*m* also encoded the chosen location with significantly shorter latencies than cOFC*l*.

We defined choice epoch as the period from 200 ms after offer 2 was presented until when choice was made via saccade and fixation on the chosen option. During this time, 18.18% (n=8/44, p=0.001, binomial test) of cOFC*m* neurons, 16.67% (n=9/54, p=0.001, binomial test) of cOFC*l* neurons, and 10.80% (n=23/213, p=0.001, binomial test) of PCC neurons encoded chosen option (offer 1 vs. 2). These proportions were not significantly different from one another (χ^2=2.62, df=2, p=0.270, Chi-square test). Encoding strength of chosen option at population level was not significantly different across regions (χ^2=1.35, p=0.510, Kruskal-Wallis test). The encoding of chosen option peaked at 90 ms in cOFC*m*, 170 ms in cOFC*l*, and 150 ms in PCC into choice epoch. These latencies were significantly different from one another (F=3.35, p=0.037, GLM with Gamma distribution). Specifically, cOFC*m* latency was significantly shorter than that in cOFC*l* (t=-2.14, p=0.033, from the same GLM fit) or PCC (t=-2.36, p=0.019, from the same GLM fit), but there was no significant difference between cOFC*l* and PCC (t=0.12, p=0.906, from the same GLM fit).

During the same choice epoch, 18.18% (n=8/44, p=0.001, binomial test) of cOFC*m* neurons, 12.96% (n=7/54, p=0.018, binomial test) of cOFC*l* neurons, and 19.25% (n=41/213, p<0.001, binomial test) of PCC neurons encoded chosen location (left vs. right). These proportions were not significantly different from one another (χ^2=1.15, df=2, p=0.562, Chi-square test). However, PCC (χ^2=5.31, df=1,

p=0.021, Chi-square test) but not cOFC*m* (χ^2=0, df=1, p=1, Chi-square test) or cOFC*l* (χ^2=0.07, df=1, p=0.787, Chi-square test) showed a higher proportion of neurons encoding chosen location than chosen option. Encoding strength of chosen location at the population level was not significantly different across the three regions (χ^2=0.20, p=0.906, Kruskal-Wallis test). The encoding of chosen location peaked around 150 ms in cOFC*m*, 230 ms in cOFC*l*, and 140 ms in PCC, into the choice epoch. These latencies were significantly different from one another (F=5.71, p=0.004, GLM with Gamma distribution). Specifically, cOFC*l* latency was significantly longer than that in cOFC*m* (t=2.36, p=0.019, from the same GLM fit) and PCC (t=3.47, p<0.001, from the same GLM fit), but there was no significant difference between those in cOFC*m* and PCC (t=0.07, p=0.944, from the same GLM fit).

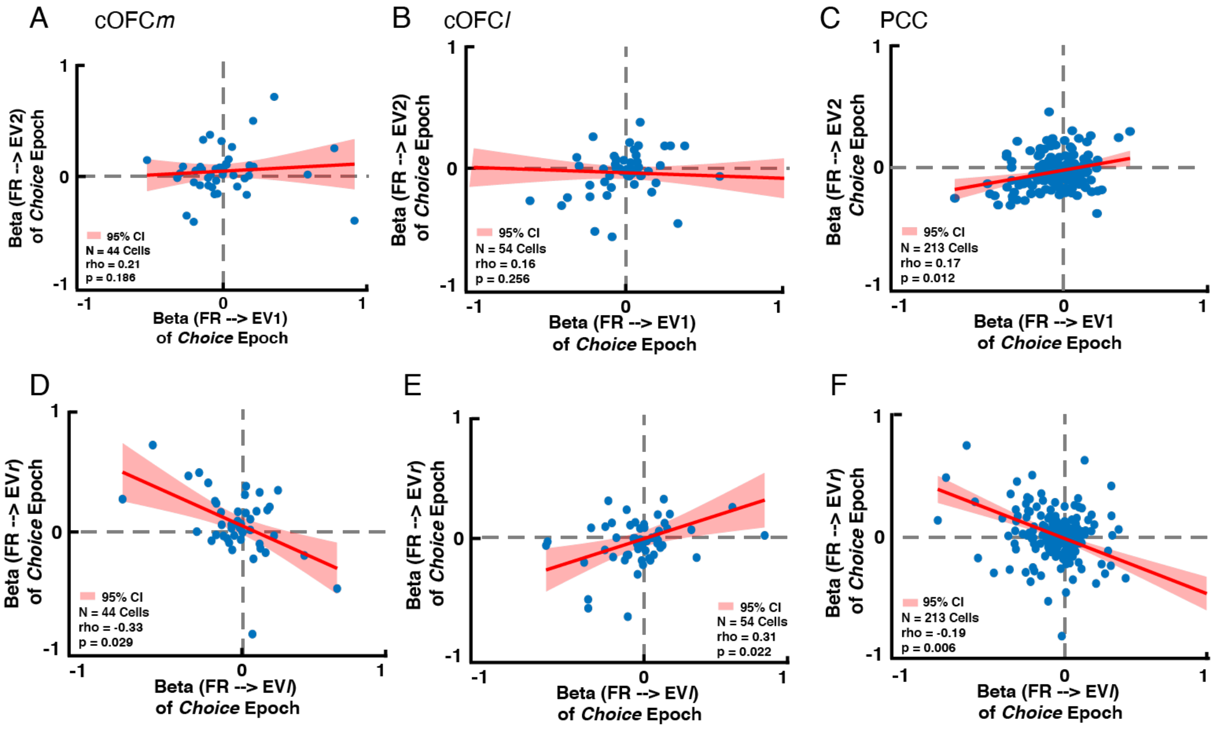

Supplementary Figure 4. Putative mutual inhibition effects. A-F: Scatter plots. Each dot represents one neuron. Solid red line: fitted Spearman correlation. Shaded area: 95% confidence interval. **A-C:** Y-axis: regression coefficient for expected value of offer 2. X-axis: regression coefficient for expected value of offer 1**. D-F:** Y-axis: regression coefficient for

expected value of right offer. X-axis: regression coefficient for expected value of left offer. A,D: cOFC***m***. B,E: cOFC***l***. C,F: PCC. These figures are complementary to Figure 3A-F (main text) in that they are results from the same analysis in a later time window (from choice epoch instead of offer 2 epoch), to show the change and development of mutual inhibition signal. The significance test used is Spearman correlation.

Robustness of findings

We verified that we had sufficient numbers of neurons in each area to perform these analyses in the following three ways:

First, we performed an outlier analysis. In the original analyses, we used Spearman correlation to examine the relationship between regression coefficients for EV1 vs. EV2 and for EVl vs. EVr, because this analysis is insensitive to outliers. This approach is more robust to outliers than the more common Pearson correlation. To further confirm that our results were not driven by any detectable outliers, we first used Cook's D to measure the global influence (both discrepancy and leverage) for each pair of regression coefficient sets in the correlation analyses in Figure 3A-F. By this method, we detected a single cell as an outlier in the analysis presented in Figure 3A. Specifically, with the outlier (as in Fig 3A), the Spearman correlation coefficient rho is -0.36 (p=0.016). After removing the outlier and repeating the same analysis, we found rho=-0.319 (p=0.038), which is also statistically significant. These two correlation coefficients are not significantly different from each other, indicating that the presence of an outlier did not itself have a measurable significant effect (z=0.208, p=0.835, Fisher's transformation test). For the analysis depicted in Figure 3C, we detected another cell as an outlier. With the outlier, the Spearman correlation coefficient rho is 0.02 (p=0.943). After removing the outlier and repeating the same analysis, we found rho=-0.019 (p=0.782). These two correlation coefficients are not significantly different (z=-0.401, p=0.689, Fisher's transformation test). These results indicate that our null finding was not driven by outliers.

Second, we used Monte-Carlo resampling to generate a 200-neuron pseudo-ensemble by randomly resampling the cOFC*m* dataset (with replacement). Then, we repeated the analyses described in the main text on the cOFC*m* pseudo-ensemble. In particular, we obtained Spearman correlation coefficients between regression weights of firing rates against the EV1 and EV2 parameters. We repeated this resampling and reanalysis 1000 times to obtain a distribution of these resampled correlation coefficients (the red distribution in Supplementary Figure 5). We take the mean of this distribution as an estimate of the true, underlying Spearman correlation coefficient. Notably, the observed Spearman correlation using the original non-resampled data was near the center of this bootstrapped distribution, and, indeed, was not significantly different from its mean. These results indicate that, despite its small sample size, our observed data were close to the distribution we would expect with a larger dataset. We

then repeated the same procedure for cOFC*l* (the orange distribution in Supplementary Figure 5). As expected, we found that the true Spearman was also not significantly different from the bootstrapped re-estimate. We repeated the same analysis for EVl vs EVr in cOFC*m* and cOFC*l*. We found that the true cOFC*m* data were not significantly different from the bootstrapped re-estimate. The Spearman correlation coefficient from recorded cOFC*l* data was slightly higher than the population estimation. However, this does not change our original finding: the distribution of the population estimation for cOFC*m* is still significantly different from that for cOFC*l* (KS stat = 0.76, p<0.001, Kolmogorov-Smirnov test). Crucially, this result confirms that the Spearman correlation coefficients for encoding formats of EVl and EVr form two significantly different distributions in cOFC*m* and cOFC*l*, suggesting that neurons from these two cOFC subregions perform significantly different neural computations for representing EVl and EVr.

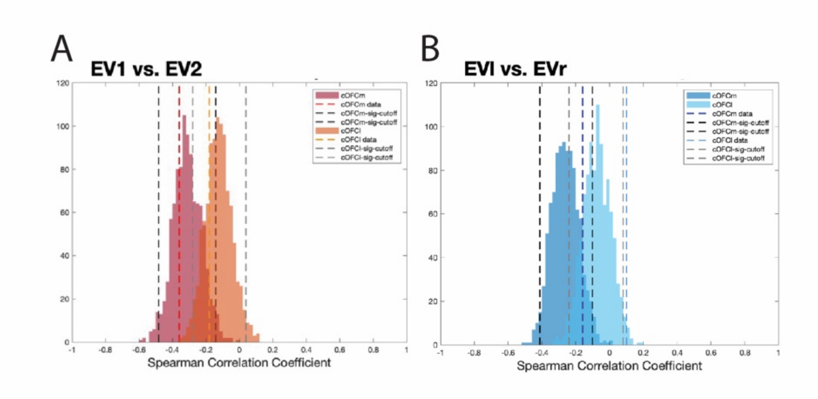

Supplementary Figure 5: Histograms showing range of values of resampled data and their overlap. A. Red distribution: distribution of correlation coefficients between regression coefficients for EV1 vs for EV2 from 1000 resampled (with repeats) cOFC***m*** ensembles. Orange distribution: same but for cOFC***l***. The cOFC***m*** distribution has a significantly more negative correlation than the cOFC***l***. B. Same as panel A, but with the EVl vs. EVr variables. Dark blue color: cOFC***m***; Light blue: cOFC***l***.

Third, we performed a power analysis. To estimate the effect size, we used the median effect size of a previous study (Wang et al., 2017) from our lab that recorded in cOFC and conducted the same ensemble analysis as in the current study. In this previous study, the median effect size of significant correlations between two sets of regression coefficients was r = 0.33 (effect sizes of the significant correlations reported in the paper: 0.68, 0.33, 0.41, 0.31, 0.27, 0.36 and 0.2). We used 0.05 as significance

level and 0.60 as power. For cOFC*m*, a power analysis with these parameters suggests that the minimum sample size required to detect an effect size of -0.36 (in Figure 3A) with significance level 0.05 and power 0.60 is n = 44. Relatedly, with a sample size of 44 neurons in cOFC*m*, significance level of 0.05, and power of 0.60, the effect size we are expecting is 0.329. Similarly, for cOFC*l*, a sample size of 54, significance level of 0.05, and power of 0.60, the effect size we are expecting is 0.298. These results indicate that our study was sufficiently powered to detect the effects that we report.

Value comparison signal in easy vs. difficult choices

We were interested in whether the cOFC*m* – PCC transformation was affected by whether a trial was easy vs difficult. To address this point, we separated correct trials into easy and difficult choice conditions. The easy condition contained trials in which the EVs of the two options were far apart; the difficult choices were those for which the EVs of the two options were close together. The median of the absolute values of the differences between the two offers served as the dividing line. As reported in the main text, the value comparison signal between EV1 and EV2 (correlation coefficient between regression coefficients for EV1 vs. EV2) in cOFC*m* Granger-caused the value comparison signal between EVl and EVr in PCC in both easy choice conditions (gc=100.75, p = 0.017) and difficult choice conditions (gc=116.51, p = 0.016). However, the Granger causal relation emerged 140 ms earlier in easy relative to difficult choice conditions. This result suggests that easy choices potentially take less time to compare and thus lead to faster transfer of choice information from cOFC*m* in a more abstract framework to PCC's more concrete, action-based framework.

Functional differences in decoding between cOFC*m*-PCC and cOFC*l* -PCC circuits

We then asked whether the relay of choice signal from value space to action space can be observed in decodability from population activities across all three regions. To answer this question, we took the normalized firing rate of each neuron over a sliding window to get the population activity pattern from all simultaneously recorded neurons in each trial. Then we trained a linear discriminant analysis (LDA) decoder on the population activity patterns from 75% of the trials and tested the decoder on the remaining 25% of the trials following a four-fold cross-validation procedure (**Methods**).

We found that at the end of offer 2 presentation (500 ms epoch), the value of the chosen option (offer 1 vs. 2) was decodable in all three of cOFC*m* (χ^2=7.41, p=0.006, chi-square test), cOFC*l* (χ^2=5.63, p=0.018), and PCC (χ^2=12.45, p<0.001), on correct trials. These three proportions were not significantly different from one another (χ^2=1.41, p=0.494), suggesting the decodability was similar across regions.

The value of the chosen options was not decodable on error trials in cOFC*m* (χ^2=0.57, p=0.448) or PCC (χ^2=0.25, p=0.613), although it was decodable in cOFC*l* (χ^2=6.83, p=0.009; **Supplementary Figure 6A**). Right before a saccade was used to select the chosen option, chosen location (left vs. right) was not decodable in cOFC*l* (χ^2=0.02, p=0.901), but was decodable in cOFC*m* (χ^2=0.25, p=0.049) and PCC (χ^2=8.85, p=0.003,), on correct trials. These three proportions were significantly different from one another (χ^2=8.37, p=0.015); the proportion in PCC was significantly higher than in cOFC*m* (χ^2=8.12, p=0.004). Chosen location was not decodable on error trials in cOFC*m* (χ^2=0.30, p=0.584), cOFC*l* (χ^2=0, p=1), or PCC (χ^2=0.06, p=0.801; **Supplementary Figure 6B**).

As a control test, we also tested decoding accuracy of EV1 high vs. low value. As shown in (**Supplementary Figure 6C-D**), both circuits / all three regions showed slightly but significantly higher than chance levels of decoding accuracy for whether EV1 was high or low in correct trials. Interestingly, decoding accuracies were not significantly different from the chance level in error trials during the offer period, and only reached slightly higher than chance level during outcome delivery.

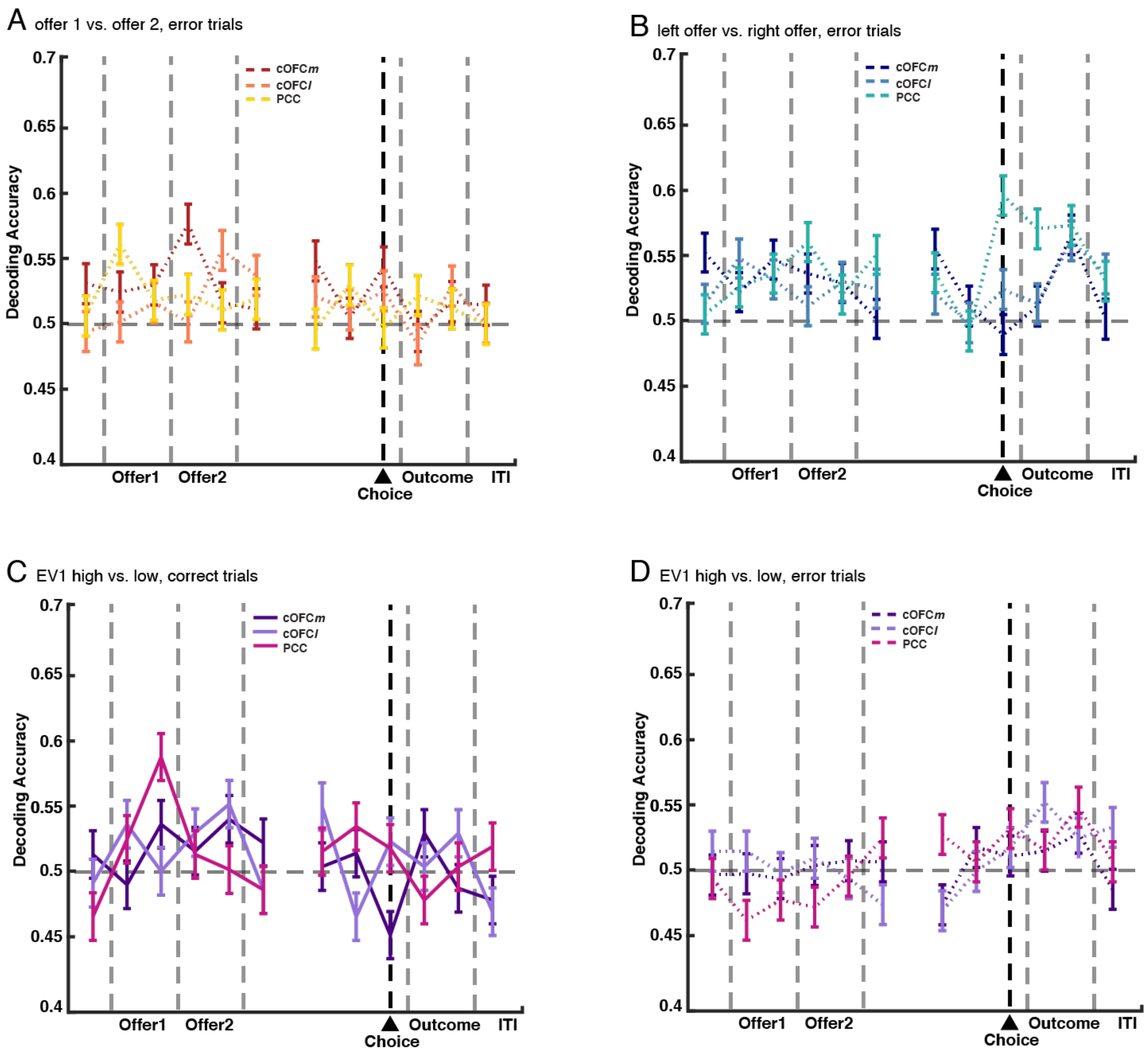

Supplementary Figure 6: Decoding accuracy. A-D: Y-axis: probability of decoding correctly. X-axis: time in a trial. Error bar: standard error of the mean. **A:** Decoding accuracy of choice option (offer 1 vs. offer 2) from error trial (choosing the offer with the smaller expected value). **B:** Decoding accuracy of choice location (left vs. right) from error trials. **C-D**: Decoding accuracy of whether the expected value of offer 1 was higher or lower than the average expected value of offer 1 from correct (**C**) and error (**D**) trials, respectively. All simultaneously recorded cells from a single region are used (with no replication) to generate trial-by-trial PCA trajectory, with which we measured the adjusted distance. A-B,D: N = 287 error trials. C: N = 805 correct trials.

Functional differences in population dynamics between cOFC*m*-PCC and cOFC*l* -PCC circuits

In contrast to the separation between trajectories for choice option (offer 1 vs. offer 2) and choice location (left vs. right), the overall distance between trial-by-trial population trajectories for high vs. low EV1 were not significantly different across cOFC*m*, cOFC*l*, and PCC (χ^2=0.20, p=0.905, Kruskal-Wallis test with Tukey-Kramer multiple comparison).

After projecting trial-by-trial population states onto the top-N PC space, we compared the adjusted distance for trial-by-trial population trajectories between correct and error trials for different pairs of task parameters. In cOFC*m*, adjusted distances between population trajectories for chosen option (offer 1 vs offer 2; χ^2=61.82, p<0.001), chosen location (left vs right; χ^2=111.99, p<0.001), and EV1 (high vs low; χ^2=120.63, p<0.001) were significantly larger in correct than in error trials. In cOFC*l* adjusted distances between population trajectories for chosen option (offer 1 vs offer 2; χ^2=29.37, p<0.001), chosen location (left vs right; χ^2=117.80, p<0.001), and EV1 (high vs low; χ^2=137.78, p<0.001) were significantly larger in correct than in error trials. Similarly, in PCC, adjusted distances between population trajectories for chosen option (offer 1vs offer 2; χ^2=93.01, p<0.001), chosen location (left vs right; χ^2=137.49, p<0.001), and EV1 (high vs low; χ^2=149.19, p<0.001) were significantly larger in correct than in error trials.

All three regions showed larger dispersion (within-condition distance; see **Methods**) in error than in correct trials. In cOFC*m*, dispersion between population trajectories for chosen option (offer 1vs2; χ^2=149.13, p<0.001, Kruskal-Wallis test), chosen location (left vs right; χ^2=149.25, p<0.001), and EV1 (high vs low; χ^2=149.25, p<0.001) were significantly larger in error than in correct trials. In cOFC*l*, dispersion between population trajectories for chosen option (offer 1vs2; χ^2=149.25, p<0.001), chosen location (left vs right; χ^2=149.25, p<0.001), and EV1 (high vs low; χ^2=149.25, p<0.001) were significantly larger in error than in correct trials. Similarly, in PCC, dispersion between population trajectories for chosen option (offer 1vs2; χ^2=149.25, p<0.001), chosen location (left vs right; χ^2=149.25, p<0.001), and EV1 (high vs low; χ^2=149.25, p<0.001) were significantly larger in error than in correct trials. Putatively, this suggest that the trial-by-trial population trajectories potentially settled in to attractor basins in correct trials but occupied more random neural subspace in error trials.

We also found that in error trials, the overall distance between trial-by-trial population trajectories for chosen option (offer 1 vs offer 2) was significantly different across cOFC*m*, cOFC*l*, and PCC (χ^2=59.88, p<0.001, Kruskal-Wallis test with Tukey-Kramer multiple comparison), with distance in cOFC*l* significantly higher than in cOFC*m* (p=0.036) and PCC (p<0.001) and distance in cOFC*m* significantly higher than in PCC (p<0.001). The overall distance in error trials between trial-by-trial population trajectories for chosen location (left vs right) was not significantly different across cOFC*m*, cOFC*l*, and PCC (χ^2=3.95, p=0.139). The overall distance between trial-by-trial population trajectories

for high vs. low EV1 were significantly different across cOFC*m*, cOFC*l*, and PCC (χ^2=8.83, p=0.012), with distance in cOFC*m* significantly higher than that in cOFC*l* (p=0.033) and PCC (p=0.023) but with no significant difference between cOFC*l* and PCC (p=0.990).

No differences between PCC gyrus and sulcus

Based on this connectivity differences observed between M1FR and M6FR, we separated recorded PCC neurons into PCC gyrus (PCCg) and PCC sulcus (PCCs) based on the placement of each recording contact. Then we conducted the ensemble analysis shown in Figures 3C and 3F on PCCg and PCCs separately, instead of on the combined PCC ensemble.

The correlation coefficient between encoding formats for EV1 and EV2 is -0.024 (p = 0.847, Spearman correlation) in PCCg, and 0.043 (p = 0.526) in PCCs. These two correlation coefficients are not significantly different from each other (z = -0.473, p = 0.637, Fisher's Transformation test). Moreover, neither the coefficient in PCCg (z = -0.320, p = 0.749) nor that in PCCs (z = 0.253, p = 0.800) was significantly different from the coefficient in the combined PCC ensemble (in Figure 3C).

The correlation coefficient between encoding formats for EVl and EVr is -0.400 ($p < 0.001$, Spearman correlation) in PCCg, and -0.151 (p = 0.024) in PCCs. These two correlation coefficients are not significantly different from each other (z = -1.934, p = 0.053, Fisher's Transformation test). Moreover, neither coefficient in PCCg (z = -1.314, p = 0.189) nor that in PCCs (z = 1.037, p = 0.300) was significantly different from the coefficient in the combined PCC ensemble (in Figure 3C).

Although we do expect functional differences between PCCs and PCCg, perhaps they would not be reflected in the particular framework studied here. In addition, the connectivity-based division identified, based on injection sites, may be too coarse. Future injections in the OFC will hopefully help to clarify the true divisions.
