# Supplementary material for: A structural and functional subdivision in central orbitofrontal cortex
Source: Nat Commun. 2022 Jun 24;13:3623. doi: 10.1038/s41467-022-31273-9 (PMC9232485; doi:10.1038/s41467-022-31273-9)
Supplement: Supplementary file 3 — Reporting Summary [file 41467_2022_31273_MOESM3_ESM.pdf]

nature portfolio

Corresponding author(s): Maya Wang

Last updated by author(s): Apr 25, 2022

Reporting Summary

Nature Portfolio wishes to improve the reproducibility of the work that we publish. This form provides structure for consistency and transparency in reporting. For further information on Nature Portfolio policies, see our Editorial Policies and the Editorial Policy Checklist.

Statistics

For all statistical analyses, confirm that the following items are present in the figure legend, table legend, main text, or Methods section.

n/a	Confirmed	
☐	☒	The exact sample size (n) for each experimental group/condition, given as a discrete number and unit of measurement
☐	☒	A statement on whether measurements were taken from distinct samples or whether the same sample was measured repeatedly
☐	☒	The statistical test(s) used AND whether they are one- or two-sided<br>*Only common tests should be described solely by name; describe more complex techniques in the Methods section.*
☐	☒	A description of all covariates tested
☐	☒	A description of any assumptions or corrections, such as tests of normality and adjustment for multiple comparisons
☐	☒	A full description of the statistical parameters including central tendency (e.g. means) or other basic estimates (e.g. regression coefficient) AND variation (e.g. standard deviation) or associated estimates of uncertainty (e.g. confidence intervals)
☐	☒	For null hypothesis testing, the test statistic (e.g. F, t, r) with confidence intervals, effect sizes, degrees of freedom and P value noted<br>*Give P values as exact values whenever suitable.*
☐	☒	For Bayesian analysis, information on the choice of priors and Markov chain Monte Carlo settings
☐	☒	For hierarchical and complex designs, identification of the appropriate level for tests and full reporting of outcomes
☐	☒	Estimates of effect sizes (e.g. Cohen's d, Pearson's r), indicating how they were calculated

Our web collection on statistics for biologists contains articles on many of the points above.

Software and code

Policy information about availability of computer code

Data collection	Anatomical data were collected using Neurolucida and Stereo Investigator software (MBF Biosciences).
Data analysis	All code was written in MATLAB R2019a and is available to readers on request to the corresponding author.

For manuscripts utilizing custom algorithms or software that are central to the research but not yet described in published literature, software must be made available to editors and reviewers. We strongly encourage code deposition in a community repository (e.g. GitHub). See the Nature Portfolio guidelines for submitting code & software for further information.

Data

Policy information about availability of data

All manuscripts must include a data availability statement. This statement should provide the following information, where applicable:

- Accession codes, unique identifiers, or web links for publicly available datasets
- A description of any restrictions on data availability
- For clinical datasets or third party data, please ensure that the statement adheres to our policy

Source data are provided with this paper. The data are available under restricted access for interpreting and verifying the research in this article. The behavioral and sorted neural data generated in this study have been deposited in the Open Science Framework (OSF) database under https://osf.io/npqhg/?view_only=7ce471770ed54e44b3caf0a7f5d0132d.

Field-specific reporting

Please select the one below that is the best fit for your research. If you are not sure, read the appropriate sections before making your selection.

- [x] Life sciences
- [ ] Behavioural & social sciences
- [ ] Ecological, evolutionary & environmental sciences

For a reference copy of the document with all sections, see nature.com/documents/nr-reporting-summary-flat.pdf

Life sciences study design

All studies must disclose on these points even when the disclosure is negative.

Sample size	N=2 for rhesus macaques is standard in the field of monkey physiology and anatomy, because of the rare nature of monkey work.
Data exclusions	No data were excluded.
Replication	Results were found in two monkeys.
Randomization	Subjects were selected in advance of the study on the basis of availability and were effectively random.
Blinding	Experimenters were necessarily blind to the conditions when they selected neurons to record from because the recorded neurons were not functionally assessed until after they were selected (on the basis of isolation properties)

Reporting for specific materials, systems and methods

We require information from authors about some types of materials, experimental systems and methods used in many studies. Here, indicate whether each material, system or method listed is relevant to your study. If you are not sure if a list item applies to your research, read the appropriate section before selecting a response.

Materials & experimental systems

n/a	Involved in the study	
[ ]	[x]	Antibodies
[x]	[ ]	Eukaryotic cell lines
[x]	[ ]	Palaeontology and archaeology
[ ]	[x]	Animals and other organisms
[x]	[ ]	Human research participants
[x]	[ ]	Clinical data
[x]	[ ]	Dual use research of concern

Methods

n/a	Involved in the study	
[x]	[ ]	ChIP-seq
[x]	[ ]	Flow cytometry
[x]	[ ]	MRI-based neuroimaging

Antibodies

Antibodies used	Rabbit tetramethylrhodamine Invitrogen (A6397)
Validation	Positive and negative control tissue samples were used to validate antibodies. Conditions for both positive and negative tissue samples included no primary and no primary/no secondary controls.

Animals and other organisms

Policy information about studies involving animals; ARRIVE guidelines recommended for reporting animal research

Laboratory animals	4 Rhesus macaques, male, 3, 4, 5 (x2) years old.
Wild animals	NA
Field-collected samples	NA
Ethics oversight	UMN IACUC protocols 1707-34950A and 2005-38127A

Note that full information on the approval of the study protocol must also be provided in the manuscript.
